# Mixed Pluronic—Cremophor Polymeric Micelles as Nanocarriers for Poorly Soluble Antibiotics—The Influence on the Antibacterial Activity

**DOI:** 10.3390/pharmaceutics13040435

**Published:** 2021-03-24

**Authors:** Maria Antonia Tănase, Adina Raducan, Petruţa Oancea, Lia Mara Diţu, Miruna Stan, Cristian Petcu, Cristina Scomoroşcenco, Claudia Mihaela Ninciuleanu, Cristina Lavinia Nistor, Ludmila Otilia Cinteza

**Affiliations:** 1 Physical Chemistry Department, University of Bucharest, 030018 Bucharest, Romania; maria.a.tanase@gmail.com (M.A.T.); adina.raducan@g.unibuc.ro (A.R.); petruta.oancea@unibuc.ro (P.O.); 2 Microbiology Department, Faculty of Biology, University of Bucharest, 60101 Bucharest, Romania; lia-mara.ditu@bio.unibuc.ro; 3 Department of Biochemistry and Molecular Biology, Faculty of Biology, ICUB-Research Institute of the University of Bucharest, University of Bucharest, 050095 Bucharest, Romania; miruna.stan@bio.unibuc.ro; 4 National Institute for Research and Development in Chemistry and Petrochemistry-ICECHIM, Polymer Department, 202 Spl. Independentei, 060021 Bucharest, Romania; cristina.scomoroscenco@icechim-pd.ro (C.S.); claudia.ninciuleanu@icechim-pd.ro (C.M.N.); cristina.nistor@icechim-pd.ro (C.L.N.)

**Keywords:** mixed polymeric micelles, drug delivery, antibiotics, Pluronic F127

## Abstract

In this work, novel polymeric mixed micelles from Pluronic F127 and Cremophor EL were investigated as drug delivery systems for Norfloxacin as model antibiotic drug. The optimal molar ratio of surfactants was determined, in order to decrease critical micellar concentration (CMC) and prepare carriers with minimal surfactant concentrations. The particle size, zeta potential, and encapsulation efficiency were determined for both pure and mixed micelles with selected composition. In vitro release kinetics of Norfloxacin from micelles show that the composition of surfactant mixture generates tunable extended release. The mixed micelles exhibit good biocompatibility against normal fibroblasts MRC-5 cells, while some cytotoxicity was found in all micellar systems at high concentrations. The influence of the surfactant components in the carrier on the antibacterial properties of Norfloxacin was investigated. The drug loaded mixed micellar formulation exhibit good activity against clinical isolated strains, compared with the CLSI recommended standard strains (*Staphylococcus aureus* ATCC 25923, *Enterococcus faecalis* ATCC 29213, *Pseudomonas aeruginosa* ATCC 27853, *Escherichia coli* ATCC 25922). *P. aeruginosa* 5399 clinical strain shows low sensitivity to Norfloxacin in all tested micelle systems. The results suggest that Cremophor EL-Pluronic F127 mixed micelles can be considered as novel controlled release delivery systems for hydrophobic antimicrobial drugs.

## 1. Introduction

Infectious disease treatments continue to impose the extensive use of antibiotics. Despite the remarkable advances made in the last century in the synthesis of new drugs with antimicrobial activity, there are many deficiencies, such as high toxicity, low solubility, reduced bioavailability, and inadequate release profile for both new and old antibiotics successfully used in the present therapies. The main issue, however, remains the multidrug resistance, which produce a huge burden on the global health system.

To overcome these drawbacks, in the last decades, nanosized drug delivery systems have been generate increasing interest of the scientific community, due to their unique physicochemical properties. The advantages of these nanoparticulate carriers in antimicrobial formulations include targeted delivery to the infection site, improved cellular internalization and drug stability, higher solubility, and sustained drug release [1]. The quinolone and their derivatives fluoroquinolone antibiotics are the most efficient class of topoisomerase inhibitors, used to treat bacterial infections caused by both Gram-positive and Gram-negative bacteria [2]. Their mechanism of action consists in the inhibition of topoisomerase enzymes (DNA gyrase implicated in genome replication and transcription), which inhibits the relaxation of supercoiled DNA and promotes the breakage of double stranded DNA [3]. Their broad antimicrobial spectrum is due to their ability to cross bacterial cell wall and cytoplasmic membranes via passive diffusion mechanism, being is strongly dependent on the lipid composition [4]. Consequently, the antibacterial activity of fluoroquinolones appears to result from the combination of efficient cellular membrane penetration and DNA gyrase inhibiting activity. Norfloxacin (1-ethyl-6-fluoro-l,4-dihydro-4-oxo-7-(1-piperazinyl)-3-quinoline carboxylic acid) is one of the most used antibiotics from the class of fluoroquinolones [5]. Due to its complex structure, it shows superior antibacterial activity against both gram positive and gram-negative bacteria, and is used to treat a large variety of respiratory or urinary tract infections.

Norfloxacin is considered as a poorly soluble drug, despite of the experimental partition coefficient log P that is reported in literature ranging from −0.43 to −1.52, quite different from the value obtained from theoretical calculation (−0.92 to 1.44) [6]. However, the large value of negative thermodynamic potential of solvation in the apolar solvent explains the poor bioavailability (less than 40%) and short half-time (3–4 h) in serum [7]. Various drug delivery systems were proposed to prolong the release, and good results have been obtained. Encapsulation in polymeric material guar gum, sodium carboxymethyl cellulose, and hydroxypropyl cellulose lead to an extended the Norfloxacin release over a period of 7–12 h [8]. A sustained release of the drug was also reported using Norfloxacin loaded proniosomes and lipid polymer hybrid nanoparticles [9].

Taking into account the advantages of different nanostructured vectors, various drug delivery systems for Norfloxacin (NFLX) have been proposed to increase its solubility, stability to chemical and photodegradation, and to improve the therapeutic benefits. For example, Ahmad et al. [10] are proposing a series of liposomal preparations of Norfloxacin with variable concentrations of phosphatidylcholine, based on the charge transfer complex between drug and phosphatidylcholine molecules. In another study, Norfloxacin-loaded nanosponges based on cyclodextrin to maximize oral absorption were proposed [11]. Norfloxacin-stearic acid solid-lipid nanoparticles were also successfully used as an oral delivery formulation [12]. Another hybrid drug delivery system containing Norfloxacin loaded into TiO_2_ nanoparticles followed by encapsulation onto poly lactic acid was found to be a suitable carrier with high antibacterial activity [13].

Nanocarriers as delivery systems for antibiotics enhance drug solubility, modulate drug release characteristics, and are also valuable tools in fighting antibiotic resistance [14]. Advanced drug delivery systems are studied for the encapsulation of norfloxacin [15] and other quinolones [16], but very rare micellar systems are investigated [17].

Micelles dispersions, from classic surfactants or polymeric surfactants, are currently used in many pharmaceutical products with various applications, from infusible solutions with chemotherapeutics or antibiotics to ocular formulations. Most of the pharmaceutical products contains as solubilizers nonionic surfactants, such as Tweens or Cremophors. Cremophor EL is FDA-approved nonionic emulsifier, used as a solubilizing agent for many years [18], produced by reacting castor oil with ethylene oxide. Therefore, it contains a mixture of unmodified castor oil and a large variety of polyethylene glycols, polyethoxylated glycerols, polyethoxylated fatty acids, and mono-, di-, and tri-esters of glycerol that are polyethoxylated to different degrees [19]. Due to its excellent emulsifying capacity, Cremophor is used for solubilization, protection, and delivery of different lipophilic active pharmaceutical ingredients (API). Numerous studies highlight Cremophor toxicity, namely anaphylactic hypersensitivity reactions, allergic shock, lipoprotein patterns and hyperlipidemia, neurotoxicity and hypotension, also at low concentration [20]. Also, some papers indicate that Cremophor can modify the toxicity profile of some active pharmaceutical ingredients [20].

Polymeric surfactants are considered a safer and more convenient alternative to classic surfactants, due to their low CMC values, good biocompatibility profile, and high drug entrapment efficiency since they possess large hydrophobic inner core. Among them, the most investigated class are polyethylene oxide-polypropylene oxide block copolymers (Pluronics or Poloxamers). These group of surfactants are FDA approved and used in various nanocarriers, in micelles, gels, and polymer stabilized nanoemulsions, to solubilize and improve the bioavailability of the poorly water-soluble active pharmaceutical ingredients. Their specific tri-block copolymer structure offers advantage regarding the modification of the release and are widely used as a carrier for controlled drug delivery [21]. Another significant advantage is that aqueous solutions of Pluronics in the presence of acids, alkalis, and metal ions are very stable [22]. Also, these polymeric micelles are more investigated because they do not show demicellization (micellar aggregate breaking) upon dilution in the presence of biological fluids such as blood and tearing.

Micellar carriers that contain Pluronic polymers exhibit good biocompatibility over a large concentration range, characteristic confirmed in many studies [23].

Most of the inconvenient of micellar systems as drug delivery systems are related to the intrinsic toxicity due to the large content of surfactant required to produce micellar aggregates even after dilution suffered after administration. In this respect, a mixture of surfactants is investigated as a possible solution to prepare mixed micelles with superior stability. When classic surfactants are added to Pluronic micelles depending on the molecular interactions taking place in the system, size and morphologies of the formed aggregates are modified [24], significantly modifying the encapsulation and release capacity of the drug. Forming mixed micelles with Pluronics is especially used to decrease the critical micellar concentration of the systems. Extensive research is available on the synergistic effects in the micellization process in mixtures containing various ionic surfactant and polymeric surfactants, while very few studies report synergism in mixed micelles of Pluronics and nonionic surfactants used in drug delivery systems formulation [25,26].

Mixed micelles are used especially to enhance the drug encapsulation and delivery parameters and have great potential as efficient drug carrier [27]. Beyond the reduction of CMC value, mixed micelles show other synergistic properties, such as increased drug loading capacity and micelle stability, higher than of the individual components. According to some reports mixed micelles that include Pluronic F127 exhibit higher solubilization capacity compared to pure F127 micelles [21]. In a study is reported a polymeric mixed micellar formulation at 1:1 Poloxamer 407/Pluronic P123 ratio, that exhibits a slower and controlled release of the drug, in contrast to the pure polymer micellar system. This behavior minimized the adverse effects associated with exceeding the safe concentration of the drug [28].

Although polymeric micelles are known to be very efficient drug carriers, little attention has been paid in using them as nanosized formulations for antibiotics. In a study carried by Khanal [29], a drug delivery system was developed by encapsulate the anionic drug cloxacillin sodium in a polyvinyl pyridine block of polystyrene-b-2-vinyl pyridine-b-ethylene oxide in order to investigate the possibility of micelles being a suitable antibiotic delivery system. Polymeric micelles were also proposed as carrier to ensure the drug transport across the blood brain barrier (BBB). Micelles prepared from cholesterol-conjugated PEG and anchored with a transactivator of transcription (TAT) peptide (TAT-PEG-b-Col) were prepared to encapsulate Ciprofloxacin and prove sustained antibacterial activity against *B. subtilis* and *E. coli*. The in vivo study evidenced that the polymeric micelles pass the BBB [30].

The aim of the present study was to prepare mixed micellar systems containing a well-known nonionic surfactant, i.e., Cremophor EL and Pluronic F127 to encapsulate a poorly soluble antibiotic Norfloxacin. The self-assembling properties in mixed surfactant systems were systematic investigated to prove the possibility of synergistic effects in micelle forming behavior. The optimization of the composition ensures the minimum surfactant amount in formulation and the use of reduced quantity of Cremophor EL, to decrease the side effects associated with it. The antibacterial activity of Norfloxacin loaded mixed micelles was evaluated, in order to consider these novel colloidal vectors as possible nanocarriers for poorly soluble antibiotics.

## 2. Materials and Methods

### 2.1. Materials

Micelle forming polymeric surfactants Pluronic^®^ F127 (BioReagent MW = 12,600 g/mol), Cremophor EL (Millipore, Burlington, MA) and model drug Norfloxacin (≥98% TLC) were purchased from Sigma-Aldrich (Merck Group, Darmstadt, Germany). Pyrene (99% purity) and the solvents absolute ethanol (99.9%), dimethyl sulfoxide (DMSO), benzene (99% purity), chloroform (99% purity), phosphate Buffer Solution (99% purity), and hydrochloric acid (36.5 g/mol, 99% purity) were also obtained from Sigma-Aldrich (Merck Group, Darmstadt, Germany). All used reagents and chemicals were used as received without further modifications.


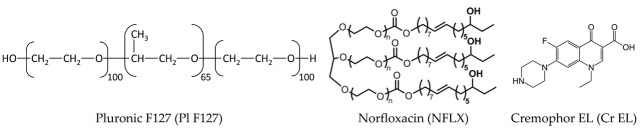


### 2.2. Preparation of Micelles

**Figure 1:**
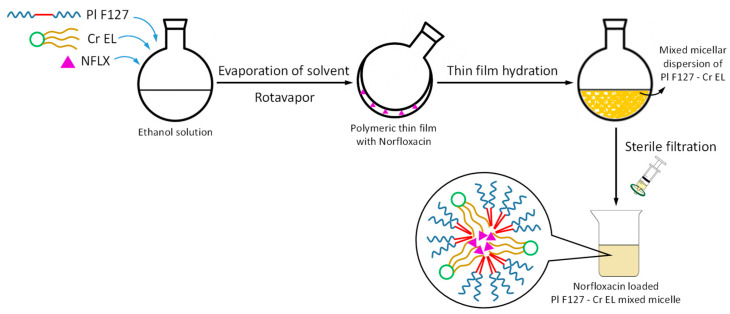
Schematic representation of the obtaining procedure for the Norfloacin loaded Pluronic F127—Cremophor EL mixed micelles.

A simple thin-film rehydration method was used to prepare micelles of Pluronic F127 and Cremophor EL. The procedure is schematically presented in the Figure 1.

Suitable amounts of surfactants or surfactant mixtures were dissolved in ethanol in a round-bottom flask. The vial was attached to a rotatory evaporator (Rotavapor-R-300^®^, Buchi Labortechnik AG, Flawil, Switzerland) and heated at 45 °C under vacuum to produce a thin film of micelle forming material deposited on the walls. After the total removal of the solvent, the required volume of distilled water was added to rehydrate the film under moderate magnetic stirring at 45 °C, for 30 min. The as prepared micellar dispersion was further filtered through sterile syringe filter Minisart^®^ 0.2 µm (Sartorius, Gottingen, Germany). For the mixed micelles, the two surfactants were added in ethanol solvent to obtain molar ratio 0.2, 0.4, 0.6 and 0.8 in the final aqueous dispersions.

The drug loaded pure and mixed micelles were prepared following the same procedure and Norfloxacin was dissolved together with the polymeric surfactants in alcohol. The dissolution of the Norfloxacin in the deposited thin film before the rehydration step ensure the maximum solubilization of the drug inside the micelle core.

### 2.3. Characterization of Mixed Micelles

*CMC* values of the pure and mixed surfactant systems were evaluated using the fluorescence method with pyrene as fluorescence probe [31]. Briefly, the fluorescence spectra of pyrene incorporated into the studied micelles (pure Cremophor EL, Pluronic F127 or mixed in various molar ratio) were recorded. A sharp variation of the slope in the graphic representation of the I3/I1 ratio (first and third vibronic peaks) indicate the CMC. For the sample preparation, pyrene was dissolved in acetone, a required volume was added in a graduate flask and the solvent was removed. Then, the surfactant solution was added to pyrene to reach a concentration of 1 × 10^−6^ M. The concentrations of the analyzed solutions ranked from 2.5 × 10^−4^ M to 2.5 × 10^−6^ M for all surfactants and their mixtures. The spectrofluorimetric measurements were performed using a Jasco FP8200 instrument (JASCO Corporation, Tokyo, Japan). The excitation wavelength for pyrene was 334 nm and its emission was recorded between 350 and 600nm. The intensity ratio between the first peak (373 nm) and the third peak (384 nm) was plotted against the concentration of the analyzed solutions and analyzed for the calculation.

Particle Size, Polydispersity Index (PI), and Zeta Potential: The pure and mixed polymeric micelles size and size distribution were measured using the dynamic light scattering (DLS) method. The data was analyzed by number and intensity weighted distributions. The zeta potential was calculated by Laser Doppler Velocimetry (LDV). The measurements were performed on a Nano ZS ZEN3600 Zetasizer (Malvern Instruments Ltd., Malvern, UK) equipment. The measurements were carried out on pristine and diluted samples, and the dilution was made with distilled water or PBS. In order to evidence the stability against aggregation and detect the presence of precipitated drug nanocrystals the samples were measured before and after filtration.

The micropolarity of the micelles was evaluated from the value of the I3/I1 ratio calculated for pyrene spectra in micellar solutions, compared to the values obtained in water and nonpolar solvent as reference.

The stability of micellar systems against dilution was evaluated by changes in size and size distribution when sample were diluted 10 time in either water or PBS. The resistance of micellar aggregates at different pH was also investigated by DLS measurements in surfactant solutions at pH = 4, pH = 6 and pH = 7.4 values.

The physicochemical stability of drug-containing micelles was evaluated by measuring the variation of the size and size distribution on samples stored at room temperature over four weeks.

### 2.4. Drug-Surfactant Interaction

The interaction of Norfloxacin with micelle forming surfactants was investigated using FTIR. The measurements were performed as follows. The spectra were recorded with a Tensor 37 Bruker equipment (Woodstock, NY, USA), using 32 scans with 4 cm^−1^ resolution in the 4000−400 cm^−1^ spectral range.

The sample pellets were prepared by adding pure components (NFLX, Pluronic or Cremophor) and drug loaded micellar solution in KBr powder, further subjected to extensive drying procedure to remove the solvent.

### 2.5. Drug Solubility and Entrapment Efficiency

For the measurement of the maximum solubility of Norfloxacin in surfactant solutions pure and mixed micelles were prepared with Cremophor EL, Pluronic F127 and binary mixture of Cremophor and Pluronic F127 with the molar ratio α = 0.2, all samples at concentrations five time their CMC value. Excess amounts (2 mg) of Norfloxacin were added in flasks and dissolved with 1 mL of ethanol. The solvent was removed in a rotaevaporator and the drug was further dissolved in 1 mL of each micellar solution. The drug—micellar systems were left to equilibrate for 12 h and the obtained dispersions were centrifuged and filtered using 0.22 µm regenerated cellulose syringe filters.

The drug amount was quantified using fluorescence method adapted in our laboratory from literature [32]. Briefly, fluorescence spectra were recorded in acidic solution of 0.1 N HCl, with λ_ex_ = 330 nm and λ_em_ = 450 nm. Concentration of Norfloxacin in micellar systems tested for calibration was: 100 µg/mL, 50 µg/mL, 25 µg/mL, 12.5 µg/mL, 6.25 µg/mL and 3.125 µg/mL. Calibration curves were obtained by plotting the fluorescence intensity at 450 nm against the concentration of Norfloxacine.

Good linearity was obtained for the concentration range 50–3.125 µg/mL for all micellar systems with R^2^ = 0.9961 for Cremophor micelles, R^2^ = 0.9903. For Pluronic F127 micelles and R^2^ = 0.9908 for the binary mixture of Cremophor and Pluronic F127 with the molar ratio, α = 0.2.

The encapsulation efficiency (EE%) was calculated as the weight ratio of encapsulated drug NFLX to the drug in feed at drug-loaded micelles:

EE (%) = Experimental drug loading/Theoretical drug loading × 100

### 2.6. In Vitro Drug Release

The release of Norfloxacin from pure micelles of Cremophor and Pluronic and mixed micelles of binary mixture of Cremophor and Pluronic at selected molar ratio α = 0.2 was studied using the dialysis bag method under physiological conditions. A volume of 2 mL of Norfloxacin encapsulated (100µg/mL) in Pluronic F127 (1.5 × 10^−3^ M), Cremophor EL (8 × 10^−4^ M) and a binary mixture of Cremophor EL and Pluronic F127 with the molar ratio, α = 0.2 (1 × 10^−3^ M) were added to the pre-swelled dialysis bag with two ends sealed with plastic sealing clips. The systems were weighted before and after adding the NFLX micellar solution. Each bag was placed in PBS release medium at room temperature, under constant stirring (50 rpm). Aliquotes of 0.5 mL were withdrawn from the release medium at predetermined time intervals (15 min, 30 min, 45 min, 1–8 h, 24 h) and replaced with the same amount of fresh medium.

All aliquots were diluted 1:10 (*v*/*v*) with 0.1N HCl and quantified by means of fluorescence spectrophotometry. The spectrofluorimetric measurements were performed using a Jasco FP8200. The excitation wavelength was 330 nm, and its emission was recorded between 350–600 nm. The intensity of the peak at 450 nm was recorded and further used to calculate the cumulative % of drug release.

The influence of the temperature and pH of the media on drug release was tested and the experiment were performed at 25 °C and 37 °C, in PBS buffer with pH values of 7.4, 6 and 4.

### 2.7. Cells Viability Assay

*Cell culture:* Human lung fibroblasts MRC-5 (ATCC CCL-171) were grown in complete Eagle’s minimal essential medium (Invitrogen, USA) containing 10% fetal bovine serum (Gibco, Carlsbad, CA, USA) at 37 °C in a humidified atmosphere with 5% CO_2_. The cells were seeded at a cell density of 5 × 10^4^ cells/cm^2^ and left to adhere for 24 h. Then, the fibroblasts were incubated for the next 24 or 48 h with different concentrations of surfactants in the range 1 × 10^−6^–1 × 10^−3^ M, which were previously sterilized by filtration with 0.2 µm pore size filter membrane. Untreated cells were used as control for all in vitro experiments.

MTT assay: The cellular viability was measured using the 3-(4,5-dimethylthiazol-2-yl)-2,5-diphenyltetrazolium bromide (MTT; Sigma-Aldrich, St. Louis, USA) assay. After 24 h of incubation, the culture medium was removed, and the cells were incubated with 1 mg/mL MTT for 2 h at 37 °C. The purple formazan crystals formed in the viable cells were dissolved with 2-propanol (Sigma-Aldrich, St. Louis, USA) and the absorbance was measured at 595 nm using a microplate reader (Flex Station, Molecular Devices).

Griess assay: The concentration of nitric oxide (NO) in the collected culture medium after the 24 h of incubation was performed with the Griess reagent, a stoichiometric solution (*v*/*v*) of 0.1% naphthylethylendiamine dihydrochloride and 1% sulphanilamide in 5% H_3_PO_4_). Increased levels of NO are related with cytotoxic effects as this molecule relates to inflammation and apoptosis. The absorbance of mix formed by equal volumes of medium supernatants and Griess reagent was read at 550 nm using the FlexStation 3 microplate reader and the NO concentration was calculated from the NaNO_2_ standard curve.

Statistical analysis: The in vitro assays were performed in triplicates and the results were presented as mean ± standard deviation (SD) of three independent experiments. The statistical significance was analyzed by Student *t*-test. A value of *p* less than 0.05 was considered significant.

### 2.8. Antibacterial Activity

The antimicrobial assays were performed using standard and clinical bacterial strains that were included in the microbial collection of University of Bucharest, Faculty of Biology, Microbiology Department: *Staphylococcus aureus* ATCC 25923 and *Staphylococcus aureus* MRSA clinical strain, *Enterococcus faecalis* ATCC 29213 and *Enterococcus faecalis* VRE clinical strain, *Pseudomonas aeruginosa* ATCC 27853 and *Pseudomonas aeruginosa* 5399 clinical strain, and *Escherichia coli* ATCC 25922 and *Escherichia coli* ESBL 135 clinical strain. To perform the experiment, two successive passages were made by passing the microbial strains on nutritious agar medium and incubating for 24 h, at 37 °C.

The qualitative screening of the anti-microbial properties was performed by an adapted spot diffusion method, according with CLSI standard (Clinical Laboratory Standard Institute, 2021). Bacterial suspensions of 1.5 × 10^8^ CFU/mL (corresponding with 0.5 McFarland standard density) obtained from 24 h microbial cultures developed on Muller Hinton agar (MHA) were used in the experiments. Petri dishes with MHA were seeded with microbial inoculums and an amount of 10 µL solution of each sample was spotted, the calculated concentration of norfloxacin being 20 µg/mL. The standard disks with 30 µg of norfloxacin were used as control for the strain’s sensitivity. The plates were left at room temperature to ensure the equal diffusion of the compound in the medium and then incubated at 37 °C for 24 h. Sensitivity was evaluated by measuring the diameters of the inhibition zones that appeared around the spot and expressed in mm.

For establishing the MIC (minimum inhibitory concentration) values of the obtained compounds we utilized a serial microdilution method performed in nutritive broth. The sterile broth was added in sterile 96 well plates and binary dilutions of each tested compound were performed in a final volume of 150 μL, starting with 20 µg/mL concentration calculated for Norfloxacin, in the first well. Further, 15 μL of microbial suspension adjusted to 1.5 × 10^7^ CFU/mL, were added in each well. The MIC values were established by spectrophotometric measurement (absorbance reading at 600 nm using BIOTEK SYNERGY-HTX ELISA multi-mode reader). Each experiment was performed in triplicate and repeated on at least three separate occasions.

Statistical analysis: For biological tests, significant differences between the means of triplicate experiments and the control were determined by using one-way ANOVA statistical analysis (significance difference was noted as * for *p* < 0.05, and ** for *p* < 0.01). All data are presented as mean values ± the standard deviations (SD).

## 3. Results and Discussion

### 3.1. Mixed Micelles Preparation and Non-Ideal Behavior in Mixed Pluronic F127-Cremophor EL Aqueous Solutions

In this study, a novel carrier from mixtures of two nonionic polymeric surfactants Cremophor EL and Pluronic F127 is proposed, in order to obtain a controlled drug delivery for Norfloxacin with extended release and higher permeability through cell membranes.

The adequate composition of the mixed polymeric micelles to ensure the minimum content of surfactants and a low value of CMC was evaluated from the non-ideal behavior of surfactant mixture, using Rubingh model. The self-assembling properties of the aqueous solution of Cremophor EL and Pluronic F127 have been studied and CMC values were calculated from the variation of pyrene fluorescence spectra, i.e, variation of the I3/I1 ratio with concentration of surfactant, as described in the previous section. The point where two linear fitting curves in premicellar (low concentration) and micellar region intersect is considered as CMC value. To evaluate the ideal or non-ideal behavior in mixed micelles, CMC theoretical values were calculated from the Clint Equation (1):

(1) $$\frac{1}{C^{*}}-\frac{\alpha_{1}}{C_{1}}+\frac{1-\alpha_{1}}{C_{2}}$$

where C* represents the CMC of the binary mixture of Surfactant 1 and 2, α is the mole fraction of surfactant 1 in the mixed solution and *C*_1_ and *C*_2_ are the individual CMCs of Surfactant 1 and Surfactant 2.

In order to calculate the intensity of interaction between the two surfactants from the binary mixture, one can use a parameter, β, calculated using Equation (2), from the model proposed by Rubingh [33]:

(2) $$\beta_{12}=\frac{\ln\frac{\alpha_{1}C^{*}}{x_{1}C_{1}}}{{\left(1-x_{1}\right)}^{2}}$$

where *α*_1_ is the mole fraction of surfactant (Cremophor EL) in the mixed micellar solution, C* is CMC of mixed micelles, *C*_1_ is the CMC of Surfactant 1 and *x*_1_ is the micellar mole fraction of Surfactant 1.

The micelle mole fraction *x*_1_ can be calculated by iteratively solving Equation (3):

(3) $$\frac{x_{2}^{1}\cdot \ln\frac{\alpha_{1}C^{*}}{x_{1}C_{1}}}{{\left(1-x_{1}\right)}^{2}\cdot \ln\frac{(1-\alpha_{1})C^{*}}{(1-x_{1})C_{1}}}=1$$

The value of parameter of interaction β quantifies the interactions between the molecules of surfactant. When attractive forces between the two surfactants are present, the value of this parameter is negative, meaning that synergism is present in the mixed micelles. Positive values for parameter β indicate an antagonistic effect, whereas in the case of β = 0 the mixed micelles formation is considered ideal. The larger the value of β (both positive or negative) the stronger the interaction (repulsion or attraction) between the two surfactants. The determination of β values allow to select mixture where synergistic behavior is present, leading to the formation of micellar aggregates at lower concentration that corresponding pure surfactants. More stable micellar systems with low surfactant content could be obtained, with a certain advantage for pharmaceutical formulation.

In Table 1, the theoretical and experimental values of CMC, together with the molar fraction in mixed micelles and parameter of interaction β are summarized for the mixture containing Cremophor EL and Pluronic F127 in various molar ratio (α is expressed relative to Cremophor EL as Surfactant 1 and Pluronic F127 as Surfactant 2).

As it is expected for a mixture with nonionic surfactants, a moderate synergistic effect is observed [25], with low values of interaction parameter, ranging from −0.22 to −1.54.

Negatives values for β were obtained for all molar fractions of 0.2, 0.6 and 0.8, but the higher value is recorded for the molar fraction 0.2, where the higher synergistic effect appears. For further experiments mixture with molar ratio α = 0.2 was selected since contain the smallest amount of Cremophor EL and the CMC value for the mixture is very low.

### 3.2. Micelles Characterization

**Figure 2:**
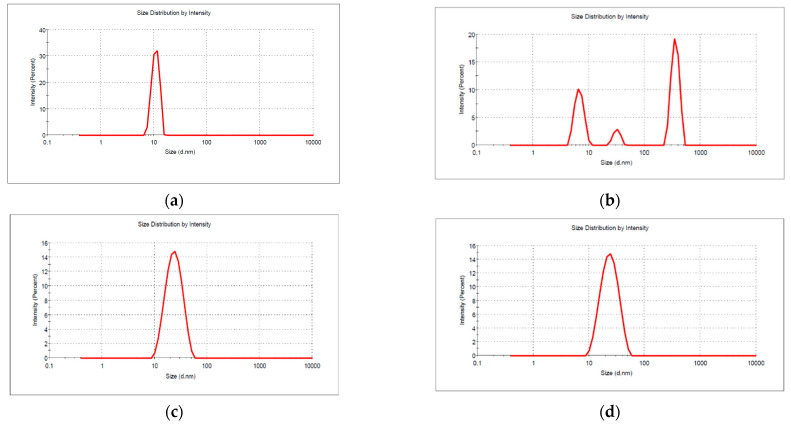
Size and size distribution of the micelles: (**a**) Cremophor EL, (**b**) Pluronic F127, (**c**) Mixed Cremophor EL-Pluronic F127 micellar system, (**d**) Norfloxacin loaded mixed Cremophor EL-Pluronic F127 micellar system.

The size and size distribution of the micelles formed in Cremophor EL, Pluronic F127, and their mixture in aqueous solution was evaluated from DLS measurements (Figure 2).

At 25 °C the DLS diagram for 6 × 10^−4^ aqueous solution of Cremophor EL shows a single population of scattering units, the surfactant micelles, with an average diameter of 12.4 ± 2.4 nm and zeta potential −0.98 ± 0.4 mV. The polydispersity index PdI is 0.157 indicating high monodispersity of the sample. In contrast, the solution of Pluronic F127 exhibits trimodal distribution, with three signals in intensity mode representation. The main diameter of the empty micelles is 31.91 ± 4.4 nm, within the range of reported values [22]. The strong intensity signal at 6.88 nm is due to the presence of the polymeric macromolecules unassociated, in equilibrium with the micellar aggregates and the signal around 354 nm is probably due to some larger micelle—micelle aggregates, which have been reported in other papers [34]. The polydispersity index of 0.669 is consistent with a trimodal distribution. The sizes of the mixed micelles range from 14.90 nm to 27.54 nm with the increase of Pluronic F127 in composition, and the presence of Pluronic non associated polymeric chain is no longer evidenced. Also, the large aggregates disappear, that confirm the increase in micellization tendency in binary mixtures as it is observed from the synergistic behavior discussed in the previous section. The size of mixed micelles is lower that is expected from an ideal mixing, and it is also consistent with the data obtained from Rubing model (Table 1), where the molar fraction of Cremophor EL in mixed micelle is always higher than the one in mixed solution.

The solubilization of the drug in mixed micelle in a concentration of 100 µg/mL produced a slightly increase in the micellar size, from 27.54 ± 8.4 nm for empty Cremophor EL—Pluronic F127 mixed micelles F127 at α = 0.2 molar ratio to 28.42 ± 7.9 nm for Norfloxacin loaded micelles. The solubilization of hydrophobic drugs inside the inner core of the micelles results in most of the case in more obvious increase of the loaded micelles [35]. Sometimes the encapsulation of the more complex molecule, such as Norfloxacin is simultaneously accompanied by a dehydration of the polymeric aggregate that produce a thinning of the hydrophilic polyoxiethylene corona [36], thus the modification of the micelle size is less evidenced.

The drug release from micelles do not produce observable changes in the size of mixed aggregates (average size before the experiment 26.42 ± 0.95 nm compared to samples after release experiment 26.90 ± 2.53 nm).

The micropolarity of the micelles was determined by using Pyrene as fluorescence probe, as recommended in literature [37]. The intensity of the vibronic bands in the fluorescence spectrum of monomeric Pyrene are reported to be very strong dependent with de polarity of the microenvironment. The ratio I3/I1 (where I1 is the first maximum emission peak at 372 nm and I3 the third one, at 384 nm) are considered as micropolarity index [38].

The I3/I1 value obtained from pyrene in water is very high, in the range 1.79–1.82, while smaller values in the range of 0.80–0.90 were determined for fluorescent probe inside micelles of various surfactants, indicating that pyrene molecules are located in a less polar environment, i.e., the hydrocarbonate core of the micellar aggregate.

For the evaluation of the microenvironmental polarity in the micelles, the ratio I3/I1 (first to third vibronic peaks) at the plateau region in the I3/I1 versus surfactant concentration curve was used, which is consistent with micellar domain.

From the spectra of pyridine recorded in pure Cremophor, pure Pluronic and mixed Cremophor-Pluronic micelles, the I3/I1 values were found 0.89, 0.96, and 0.90, respectively. The value of I3/I1 ratio of 0.89 for the Cremophor micellar system is consistent with the chemical structure of surfactant, which can produce a nonpolar core resembling to the hydrocarbon media to accommodate pyrene molecules. In contrast, the higher value, 0.96 found for Pluronic F127 micelles suggests that these micelles provide a microenvironment for the pyrene probe more polar than usual surfactants (in the domain of moderate polarity), due to the tendency of water penetration at the core-corona border [37]. The mixed Cremophor-Pluronic micelle exhibits an I3/I1 value similar to the pure Cremophor micelles, probably due to the higher molar ratio of Cremophor inside the mixed micelles than the actual molar ratio in the bulk surfactant solution.

The resistance of the micelles against dilution and pH changes was also checked.

The samples of pure and mixed micelles diluted 10 times show similar values of surfactant aggregates after dilution compared to concentrated ones. For drug loaded Cremophor EL-Pluronic F127 mixed micelles at α = 0.2, for example, average size of concentrated dispersion (1 × 10^−3^ M) in PBS was 26.41 ± 0.95 nm while after a tenfold dilution, the size was 26.33 ± 0.95 nm.

The pH value of the dispersion media is expected to show negligible influence on the micellar size and shape, since both surfactants are nonionic. Thus, the values of the average size of mixed micelles Cremophor EL-Pluronic F127 at α = 0.2 are found 26.97 ± 1.41 nm at pH = 4, 26.94 ± 2.54 at pH = 6, and 26.41 ± 0.95 nm at pH = 7.4, respectively. The mixed micelles prove to be resistant to dilution and no effect of pH variation in the range 4–7.4 affect the aggregation.

The DLS measurements were also applied to the samples after four weeks storage at room temperature. No significant changes were recorded in the size and size distribution of the void and NFLX.

### 3.3. Drug—Polymeric Micelle Interactions

**Figure 3:**
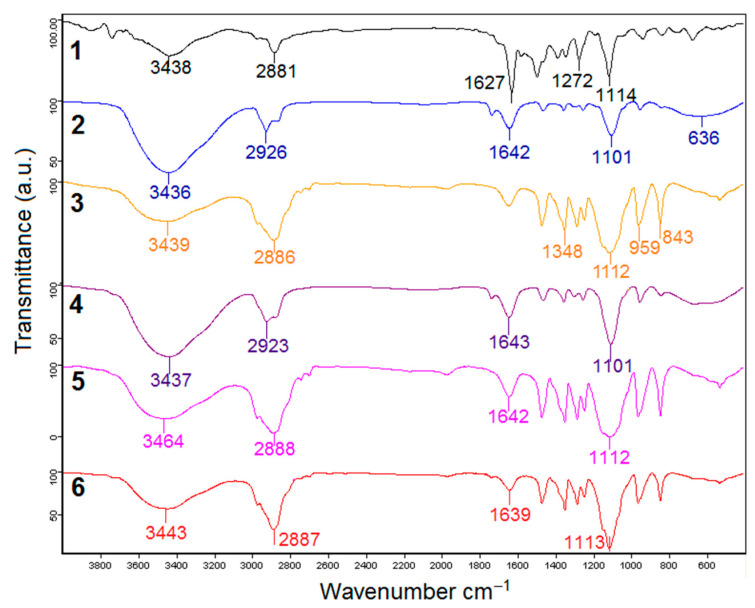
FTIR spectra of (1) Norfloxacin, (2) Cremophor EL, (3) Pluronic F127, (4) Norfloxacin in Cremophor EL micelles 100 µg/mL, (5) Norfloxacin in mixed micelles Cremophor EL-Pluronic F127 and (6) Norfloxacin in Pluronic F127 micelles 100 µg/mL.

To investigate the interaction of Norfloxacin molecules with the polymeric surfactants in micelles Fourier transformed infrared spectroscopy were used. In Figure 3, the FTIR spectra of pure Norfloxacin (1), Cremophor EL (2), Pluronic F127 (3), Norfloxacin in Cremophor EL micelles 100 µg/mL (4), Norfloxacin in mixed micelles Cremophor EL-Pluronic F127 (5) and Norfloxacin in Pluronic F127 micelles 100 µg/mL (6) are displayed.

The FTIR spectrum of Norfloxacin shows specific absorption peaks, similar to those reported in literature [13] as follows: the wide band centered at 3438 cm^−1^ is produced by both imino moiety of piperazinyl groups (–NH stretching vibration) and –OH group from acid and the band at 2881 cm^−1^ correspond to C–H stretching vibrations. The absorptions at 1627 cm^−1^ are characteristic for quinolones (–NH bending vibration) and the region between 1500–1450 cm^−1^ for =O–C–O– group of acid (υ_s_ stretching vibration). The bending vibration of –OH was found at 1272 cm^−1^ and the strong absorption at 1114 cm^−1^ was related to C–F group.

For Cremophor EL were registered a broad band at 3436 cm^−1^ for the –OH group, the band at 2926 cm^−1^ attributed to C-H stretch and the very small absorption band between 1715–1730 cm^−1^ characteristic to C=O stretch for esters. The stretching band of C=C was found at 1642 cm^−1^, the band from 1101 cm^−1^ was attributed to C-O stretch from alcohols and the wide absorption of 636 cm^−1^ to =C-H bend, similar to reported data in the literature [39]. In the spectra of Pluronic F127 micellar systems specific signal were observed as broad band at 3439 cm^−1^ for the –OH group, the bend of 2886 cm^−1^ was attributed to C-H stretch, 1348 cm^–1^ (in-plane O-H bend) and 1112 cm^–1^ (C-O stretch) [40].

For the sample with Norfloxacin in Cremophor EL micelles, FTIR spectrum absorptions at 2923 cm^−1^ attributed to C-H stretch, 1643 cm^−1^, stretching band of C=C, 1101 cm^−1^ attributed C-O stretch from alcohols (identical to that of Cremophor), 950 cm^−1^ attributed to *trans*-CH=CH- group and 669 cm^−1^ attributed to =C-H bend. The most evident change is the broadening and increase intensity of the absorption band at 3438 cm^−1^, probably due to the formation of numerous hydrogen bonding with OH group from acid (Norfloxacin) and from alcohol (Cremophor) and other interactions with the imino moiety of piperazinyl groups (NH stretching vibration).

As a result of the encapsulation of the Norfloxacin in Pluronic F127 micelles no significant changes were observed in the peak intensities and positions, with the exception of the wide absorption band in the 3500 cm^−1^ region, which is wider and more intense, due to the formation of hydrogen bonds between drug and polymer molecules. In the spectra of encapsulated Norfloxacin in the mixed micelles changes in shape and position of peaks could not be observed, except the same broadening and shifting to higher wavenumber of the peak at 3438 cm^−1^ to 3464 cm^−1^ and a minor shift to higher wavenumber of the peak corresponding to C-F bending. These changes are due to the interactions of the Norfloxacin with Cremophor and Pluronic F127, as a result of encapsulation of the Norfloxacin in the mixed micelles.

### 3.4. Drug Solubility and Encapsulation Efficiency

The maximum solubility of NFLX in micelles and encapsulation efficiency are tabulated in Table 2.

The concentration in the micellar dispersion were selected to represent 20 time the CMC value, in order to compare the solubility in micelle aggregates rather than conventional procedure versus weight of the polymeric material. The drug loading capacity expressed as % of NFLX from the weight of drug and micelle forming materials is 5% for Cremohor EL, 4.67% for Pluronic F127 and 4.89% for mixed micelles Cremohor EL-Pluronic F127 with molar ration α = 0.2 (thus low content of Cremophor). One can conclude that a high loading capacity is maintained in mixed micelles, even at a significantly decreased surfactant concentration at the molar ratio where the synergistic effect is present, due to the favored micellization process.

### 3.5. In Vitro Drug Release

**Figure 4:**
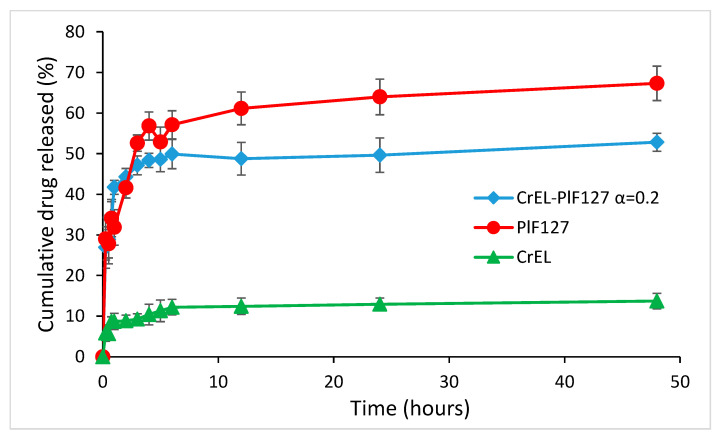
In vitro drug release profile for NFLX in Cremohor EL, Pluronic F127 and CrEL-Pl F127 (α = 0.2) mixed micelles.

The release kinetics of Norfloxacin from various micellar dispersions was evaluated using PBS as receiving media and the results are presented in Figure 4.

The drug release profile is significantly dependent to the composition of micelles. Norfloxacin encapsulated in Cremohor EL micelles exhibit a cumulative release less than 20% up to 48 h, probably due to the compact packing of the hydrophobic chains of the surfactant inside the inner core and higher tendency of drug retention. The release profile show a burst in the first hour, then a decrease in the release rate. The burst region is present also in the Pluronic F127 micellar dispersion for the first 2.5 h, but cumulative release is far more important, up to 64%. The Norfloxacin molecules could be located both in the hydrophobic inner region of polypropylene oxide (PPO) and embedded in the hydrophilic corona of polyoxyethylene (POE) groups in the Pluronic micellar aggregate and lead to a higher extent of drug released than from Cremophor EL micelles. The release from the mixed micelles retains the burst segment, less pronounced compared to the situation in Cremophor micelles, due to the small amount of this polymeric surfactant in α = 0.2 selected composition. The cumulative release is improved compared to Cremophor EL micelles, due to the influence of the large Pluronic, content up to 49% at 48 h.

**Figure 5:**
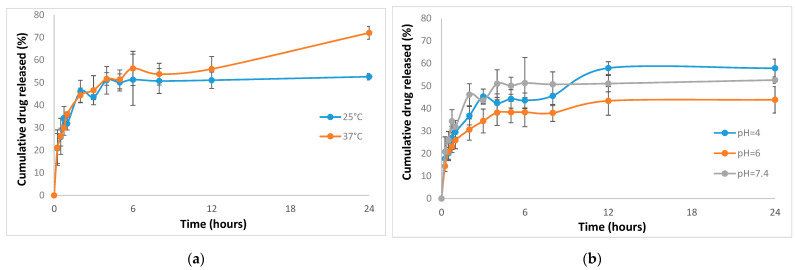
In vitro drug release profile for NFLX in mixed CrEL-Pl F127 (α = 0.2) micelles at various temperature (**a**) and pH values (**b**).

The influence of the temperature and pH on the release profile of NFLX in selected mixed micelles (Cremophor-Pluronic F127 at molar ratio 0.2) is presented in Figure 5.

Since both Cremophor EL and Pluronic F127 are nonionic surfactants the effect of temperature up to 50 °C is negligible on the size and morphology of the micellar aggregates, thus the release of Norfloxacin encapsulates in either pure or mixed micelles is not significantly affected by the increase of the temperature from 25 °C to 37 °C. The kinetic profile is similar for the two temperatures investigated up to 12 h, with a moderate increase (from 51.8% at 25 °C to 72.0% at 37 °C) at 24 h, probably due to the increase in the aqueous solubility of drug when rise the temperature.

The release profile at various pH values shows an increase of the cumulative drug release at 24 h up to 60.80% at pH = 4, while at the pH = 6 a decrease of the total NFLX release to 47% is observed. This unexpected variation could be the result of the complex equilibrium of the drug species inside the micelles and release media, due to the peculiar variation of the solubility of Norfloxacin with pH.

This behavior is not the result of changes in micelle properties, but of the intrinsic properties of the drug. Size and size distribution of the Cremophor-Pluronic F127 (α = 0.2) micelles mixed micelles, as well as those for pure Cremophor or Pluronic micelles is not affected by the modification of the pH, according to the DLS measurements presented in Section 3.2.

The chemical structure of Norfloxacin produces amphiphilic, ionized and neutral species with variation of the pH, i.e at pH = 7.4 existence of both neutral NFLX^0^ and zwitterionic NFLX^±^ was evidenced, at pH = 6 a quasi-equimolecular mixture of zwitterionic NFLX^±^ and ionic NFLX^+^ is present and at pH = 4 only the ionic NFLX^+^ is observed. This ionization behavior is consistent with the dramatic changes in solubility of NFLX with the pH, from 0.3 M at pH = 5 to 2.9 × 10^−3^ M at pH = 6 and 1.3 × 10^−3^ M at pH = 7 [41].

From the significant increase of the aqueous solubility of Norfloxacin at pH = 4, one can expect a more obvious increase in the drug released. However, the fully charged species NFLX^+^ is stronger retained inside the micelles due to the interaction with the –OH and –COOH groups from the Pluronic and Cremophor molecules. At pH = 6 the coexistence of both zwitterionic and protonated Norfloxacin species results in a lower content of drug released.

The combination of the two polymeric surfactants results in a sustained release of NFLX form micelles that can ensure a long-term delivery of the antibiotic.

### 3.6. Biocompatibility of Mixed Micelles

**Figure 6:**
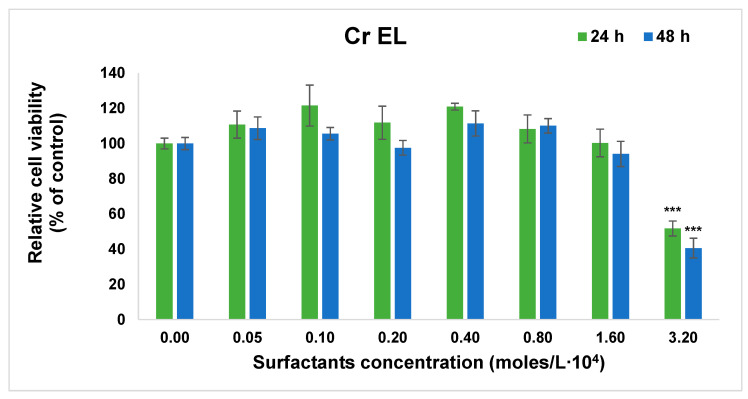

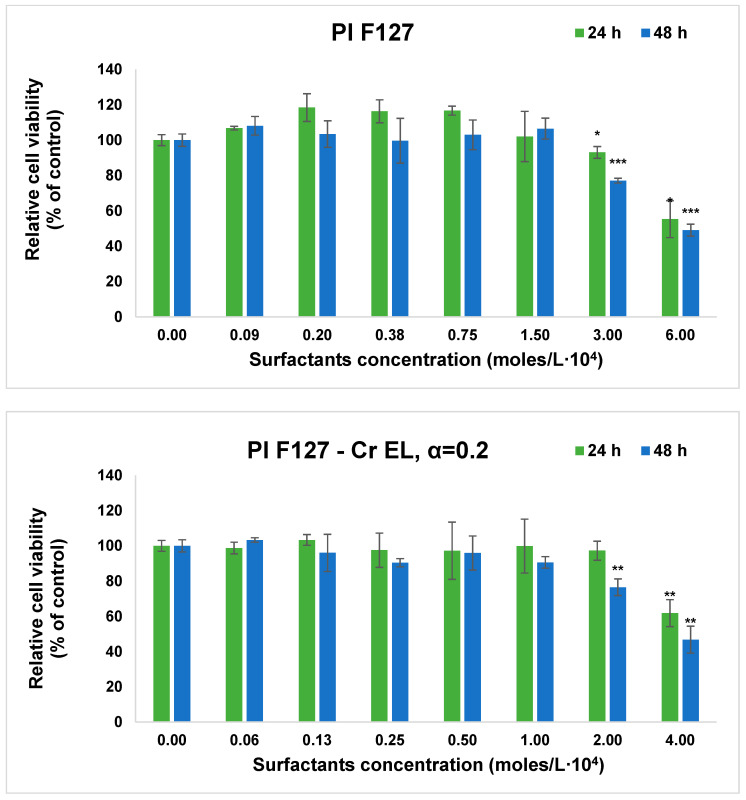
Cell viability results obtained by MTT assay after 24 and 48 h of cell growth in the presence of surfactants. Results are presented as mean ± standard deviation of three independent experiments (* *p* < 0.05, ** *p* < 0.01 and *** *p* < 0.001 compared with control).

**Figure 7:**
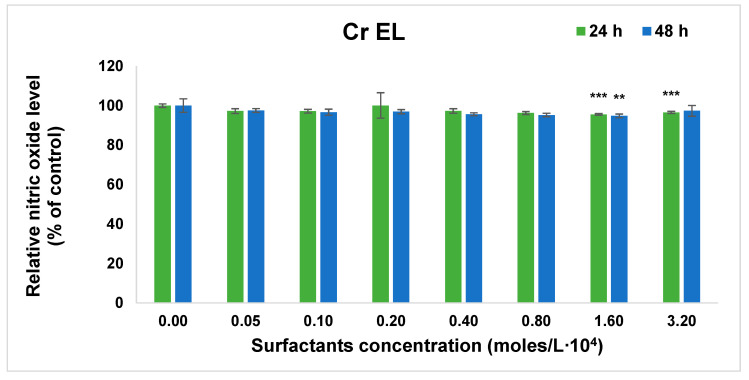

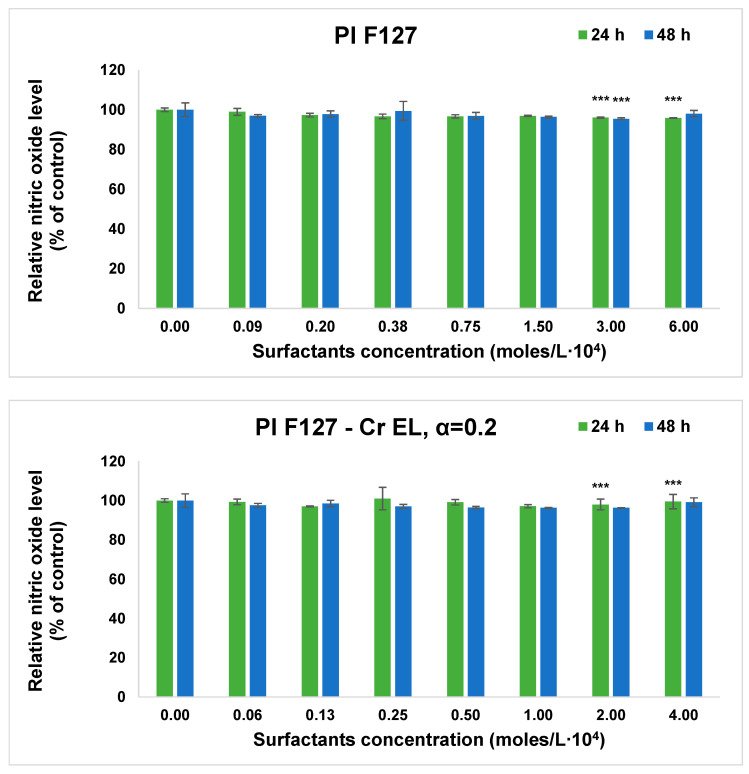
NO release measured by Griess assay after 24 and 48 h of cell growth in the presence of surfactants. Results are presented as mean ± standard deviation of three independent experiments (** *p* < 0.01 and *** *p* < 0.001 compared with control).

The effect of surfactants on normal cells was studied in terms of cell viability by MTT assay and cellular inflammation and membrane damage with nitric oxide (NO) release test. The study was performed on human lung fibroblasts MRC-5 cells selected as model normal cells with moderate sensibility to dispersions. The results are shown in Figure 6 and Figure 7.

As shown in Figure 6, the Cremophor EL micellar solution in the range 5 × 10^−6^–2 × 10^−4^ M did not affect the number of viable cells after 24 h of incubation compared with the control. Although no change in cell viability was measured for sample Cremophor EL even at concentration approximative 5 times higher the CMC, a significant decrease was noticed for the 3.2 × 10^−4^ M concentration, after both time intervals of incubation with surfactants.

The micellar dispersions based on Pluronic F127 show lack of cytotoxicity up to 1.5 × 10^−4^ M, as it is reported in most paper for low concentrated polymeric solutions below 8 × 10^−6^ M, in premicellar region [42]. An unexpected dose-dependent decrease was obtained for Pluronic concentrated micellar disperions from 3 × 10^−4^ M to 6 × 10^−4^ M, where the cellular viability decreased to 49% compared to control value after 48 h of exposure.

In the mixed micelle system with molar ratio Cremophor EL- Pluronic F127 α = 0.2, the dose dependent variation of the cytotoxicity is observed similar to Pluronic F127 micellar dispersion, probably due to the small amount of the Cremophor in the mixture. Significant decrease in the cellular viability is recorded after 24 and 48 h at higher concentration than 2 × 10^−4^ M of mixed surfactants.

Since the synergistic molar ratio α = 0.2 exhibits the low value of CMC 4 × 10^−5^ M, solutions with concentration 3-fold CMC will ensure the existence of the micellar aggregates and remains in the region without evident toxicity.

The amount of NO released in the culture medium was assessed as a valuable indicator of inflammation produced by the contact with surfactant solutions in premicellar and micellar regions. As it is shown in Figure 7, no significant changes in NO release after cell exposure to surfactants over whole range of studied concentrations could be observed. Thus, it was concluded that even in the case of the Cremohore EL presence, the surfactant micelles did not induce inflammation in human fibroblast cell cultures.

### 3.7. Antibacterial Activity

**Figure 8:**
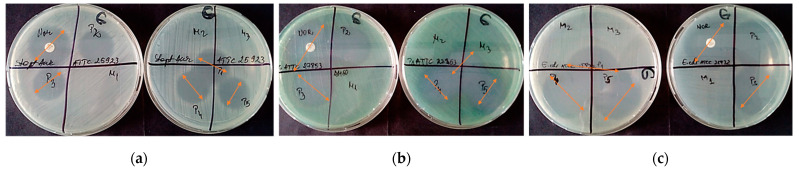
Aspect of the inhibition zones for microorganisms (**a**) *Staphylococcus aureus* ATCC 25923, (**b**) *Pseudomonas aeruginosa* ATCC 27853 and (**c**) *Escherichia coli* ATCC 25922. The samples are denoted P1 = NFLX in water; P2 = NFLX in DMSO; P3 = NFLX in Cremophor EL micellar solution; P4 = NFLX in Pluronic F127 micellar solution; P5 = NFLX in mixed micellar solution, while M1, M2 and M3 are empty micelles of Cremophor EL, Pluronic F127 and mixed micelles, respectively.

The qualitative screening of the antimicrobial activity evaluated the efficiency of the Norfloxacin encapsulated in Cremophor EL and Pluronic F127 micellar carriers, by measuring the diameters of the inhibition zone expressed by each tested bacterial strain. The value of the diameters of the inhibition zones demonstrated the achievement of a concentration gradient around the spot following the release of the antibiotic from the micellar carrier (20 µg in 10 µL micellar solution), by comparison with the concentration gradient achieved by the release of antibiotics from the standard disc (30 µg/disk) (Figure 8).

It cannot be observed significant difference between Cremophor and Pluronic micellar carriers (P3 and P4) or combination of them (P5) on the growth of standard bacterial strains.

**Figure 9:**
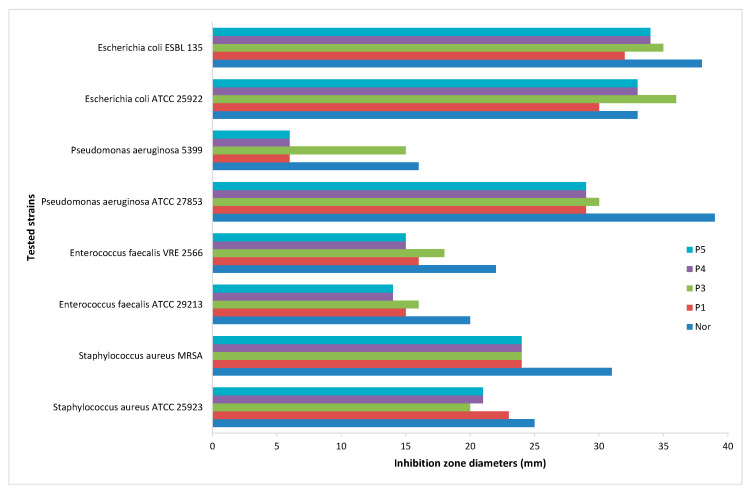
Graphic representation of inhibition zone diameters (mm).

In Figure 9 the values of the diameter of inhibition zone recorded for both clinical and standard bacterial strains are presented.

As shown in Figure 9, different behaviors for the clinical and the standard bacterial strains exposed to NFLX are recorded, with the clinical strains expressing larger diameters of the inhibition zones in both cases standard disc and micellar carriers. The exception is the *P. aeruginosa* 5399 clinical strain which shows low sensitivity to Norfloxacin in all the tested systems. It seems that this bacterial strain is resistant to fluoroquinolone, given the diameter of the inhibition zone for the norfloxacin (16 mm < 18 mm, according with CLSI 2021) and by using the micellar carrier for antibiotic the sensitivity pattern has not changed when NFLX was encapsulated in Cremophor and mixed micelles compared to NFLX in aqueous solution. A rather higher sensibility is recorded to the NFLX encapsulated in Pluronic F127 (15 mm), probably due to the cellular membrane permeability induced by the tri-block copolymer micelles.

For the *E. coli* strains, both standard and clinical strains, the diameter of inhibition zones produced by NFLX encapsulated in micelles were larger than the antibiotic in aqueous solution.

**Figure 10:**
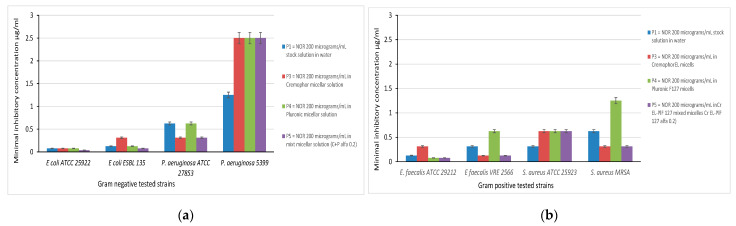
Graphic representation of minimal inhibitory concentration values for: (**a**) Gram negative tested strains and (**b**) Gram positive tested strains.

The quantitative results expressed by minimal inhibitory concentration values allowed the quantitative evaluation of the carriers’ efficiency in terms of antibiotic release capacity in the liquid medium (Figure 10).

For *E. coli* ATCC 25922 strains the minimal inhibitory concentrations were similar for the reference aqueous solution of NFLX and the drug encapsulated in various micellar systems, with the exception of NFLX in mixed Cremophor EL- Pluronic F127 micelles, which exhibited a lower MIC of 0.039 µg/mL compared to 0.078 µg/mL. The clinical strain *E. coli* is less susceptible to NFLX encapsulated in Cremophor EL (MIC of 0.31 µg/mL) and again the highest activity was observed for the formulation with mixed surfactants (MIC 0.078 µg/mL).

In the case of *P. aeruginosa* ATCC 27853, a similar efficiency was observed for NFLX in aqueous solution and Pluronic micelles (MIC 0.625 µg/mL), while the presence of Cremophor EL in pure and mixed micelles reduced the MIC value to 0.312 µg/mL. As observed in the qualitative test, *P. aeruginosa* clinical strain proved to be more resistant to NFLX, and the encapsulation in micelles leads to an unexpected increase of the MIC values for all surfactants.

In the case of the Gram-positive microorganisms there were also differences in the results recorded for standard and clinical strains depending on the composition of the micellar carrier. For standard *E. fecalis* ATCC 29212 the lowest efficiency was demonstrated by the NFLX loaded Cremophor EL micelle dispersion (MIC value 0.312 µg/mL), while the encapsulation in Pluronic F127 and in the mixed micelles reduced the MIC value to quarter. The clinical *E. fecalis* VRE 2566 strain developed a high value of MIC in the case of NFLX in Pluronic micelles (MIC value 0.625 µg/mL). Again, the encapsulation in mixed micelles decreased the value of MIC up to 0.125 µg/mL.

For *S. aureus* standard strain ATCC 25923 the MIC values for NFLX in all micellar systems proved to be double compared to the value recorded for aqueous solution (0.625 µg/mL compared to 0.312 µg/mL).

In the case of resistant *S. aureus* strain, no significant improvement was observed with the Pluronic F127 micelles, on the contrary the MIC value obtained is the higher obtained in our study (1.25 µg/mL) for Gram positive microbial cultures. The presence of Cremohor EL as pure micelles or in combination with Pluronic F127 increased the antibacterial efficiency of the encapsulated drug.

The micelles of two polymeric surfactants used in this study had different effects on the Norfloxacin antibacterial efficiency on different microorganisms, and a variation in behavior could also observed between standard and clinical strains. A possible explanation is the specific interaction between the cellular membrane and micelle forming surfactants, that should be investigated in detail in order design drug delivery systems for NFLX with enhanced activity against resistant microbial strains.

Pluronic polymeric surfactants have begun to be investigated to elucidate their role in management of biofilm formation, but relevant data and explanations about the complex interactions with the microbial cellular membrane are still lacking [43].

## 4. Conclusions

Polymeric micelles were prepared as drug delivery systems for model poorly soluble antibiotic Norfloxacin in order to achieve better stability and controlled delivery of drug. Two polymeric surfactants Cremophor EL and POE-PPO-POE triblock copolymer Pluronic F127 and their mixtures in various molar ratio were studied. The non-ideal behavior of the self-assembling process in surfactant mixtures reveal the existence of synergistic effects over the whole range of composition, with a maximum value of interaction parameter in mixed micelle for the molar ratio α = 0.2. The micelles were characterized in term of size, size distribution and drug solubilization, and the selected composition with low content of Cremophor EL show suitable performance to be used as Norfloxacin carrier.

The mixed micelles selected exhibit resistance against dilution and pH changes in the range of 4–7.4. The encapsulation efficiency of NFLX in mixed CrEL—Pl F127 micelles was 52.2 ± 2.1% and do not significantly change the size of nanocarrier after the drug encapsulation.

The drug release profile exhibited an initial burst (more obvious in Cremophor EL micelles) in all micellar dispersion, with a cumulative release up to 51.8% in mixed Cremophor EL—Pluronic F127 system at 25 °C, intermediate from the value obtained for Cremphor EL and Pluronic F127 pure micelles. The total amount released in 24 h was increased to 72% at temperature of 37 °C. The results of cytotoxicity and inflammation tests on normal fibroblast cells indicate that both studied surfactants and their mixtures prove high biocompatibility for concentrations below 2–3 × 10^−4^ M.

The analysis of qualitative and quantitative results showed an improvement of the antibacterial properties of Norfloxacin against the majority of the tested bacterial strains, both Gram positive and Gram negative, when the polymeric micelles were used as carriers.

The sample with NFLX encapsulated in the novel carrier prove to be effective on both *E. coli* ATCC 25922 and clinical strains, with MIC value of 0.039 µg/mL and 0.078 µg/mL, respectively. The minimum inhibitory concentrations were found to vary greatly with nature and provenience of the microbial strain and with the surfactant composition of the formulation. As a general conclusion, the drug loaded mixed micellar formulation exhibits good activity against clinical isolated strains, compared with the CLSI recommended standard strains, thus Cremophor EL-Pluronic F127 mixed micelles can be considered as novel controlled release delivery systems for hydrophobic antimicrobial drugs.

## Figures and Tables

**Table 1:** The micellization parameters in binary mixtures Cremophor EL and Pluronic F127 in various molar ratio obtained from the Rubingh model.

A	Experimental CMC * (M)	Calculated CMC * (M)	X	β
0	8 × 10^−5^	-	-	-
0.2	4 × 10^−5^	6.0 × 10^−5^	0.46	−1.54
0.4	4.5 × 10^−5^	4.8 × 10^−5^	0.57	−0.82
0.6	3.0 × 10^−5^	4.0 × 10^−5^	0.69	−0.45
0.8	2.8 × 10^−5^	3.4 × 10^−5^	0.83	−0.22
1	3 × 10^−5^	-	-	-

* using Equation (1).

**Table 2:** Amount of Norfloxacin (NFLX) solubilized in polymeric micelles and encapsulation efficiency at 25 °C.

Micellar Dispersion	NFL Solubilized (µg/mL)	EE (%)
Cremohor EL	99.2	48.5 ± 3.3
Pluronic F127	93.5	45.7 ± 1.5
Mixed CrEL-Pl F127	93.8	52.2 ± 2.1

## Data Availability

Not Applicable.
